# EpicCapo: epitope prediction using combined information of amino acid pairwise contact potentials and HLA-peptide contact site information

**DOI:** 10.1186/1471-2105-13-313

**Published:** 2012-11-24

**Authors:** Thammakorn Saethang, Osamu Hirose, Ingorn Kimkong, Vu Anh Tran, Xuan Tho Dang, Lan Anh T Nguyen, Tu Kien T Le, Mamoru Kubo, Yoichi Yamada, Kenji Satou

**Affiliations:** 1 Graduate School of Natural Science and Technology, Kanazawa University, Kanazawa, Japan; 2 Institute of Science and Engineering, Kanazawa University, Kanazawa, Japan; 3 Department of Microbiology, Faculty of Science, Kasetsart University, Bangkok, Thailand

## Abstract

**Background:**

Epitope identification is an essential step toward synthetic vaccine development since epitopes play an important role in activating immune response. Classical experimental approaches are laborious and time-consuming, and therefore computational methods for generating epitope candidates have been actively studied. Most of these methods, however, are based on sophisticated nonlinear techniques for achieving higher predictive performance. The use of these techniques tend to diminish their interpretability with respect to binding potential: that is, they do not provide much insight into binding mechanisms.

**Results:**

We have developed a novel epitope prediction method named EpicCapo and its variants, EpicCapo^+^ and EpicCapo^+REF^. Nonapeptides were encoded numerically using a novel peptide-encoding scheme for machine learning algorithms by utilizing 40 amino acid pairwise contact potentials (referred to as AAPPs throughout this paper). The predictive performances of EpicCapo^+^ and EpicCapo^+REF^ outperformed other state-of-the-art methods without losing interpretability. Interestingly, the most informative AAPPs estimated by our study were those developed by Micheletti and Simons while previous studies utilized two AAPPs developed by Miyazawa & Jernigan and Betancourt & Thirumalai. In addition, we found that all amino acid positions in nonapeptides could effect on performances of the predictive models including non-anchor positions. Finally, EpicCapo^+REF^ was applied to identify candidates of promiscuous epitopes. As a result, 67.1% of the predicted nonapeptides epitopes were consistent with preceding studies based on immunological experiments.

**Conclusions:**

Our method achieved high performance in testing with benchmark datasets. In addition, our study identified a number of candidates of promiscuous CTL epitopes consistent with previously reported immunological experiments. We speculate that our techniques may be useful in the development of new vaccines. The R implementation of EpicCapo^+REF^ is available at http://pirun.ku.ac.th/~fsciiok/EpicCapoREF.zip. Datasets are available at http://pirun.ku.ac.th/~fsciiok/Datasets.zip.

## Background

Cytotoxic T lymphocytes (CTLs) play an important role in the vertebrate immune system. CTLs recognize pathogens via peptide presentation on major histocompatibility complex molecules (MHCs). If the source of peptides is an infectious virus, the CTL response could be stimulated, thus leading to the elimination of virus-infected cells [1]. MHC-bound peptides are called epitopes, and they are usually composed of 8–20 amino acids. Epitope identification is an essential step toward synthetic vaccine development, since epitopes play an important role in the activation of the immune response [2]. Epitopes are traditionally identified by synthesizing a large number of nonapeptides and subsequently performing affinity assays. Those peptides with high affinity for MHC proteins are considered as potential epitopes. However, the process of developing a new vaccine is time-consuming and laborious when performed with traditional methods. To avoid the problems of such bottlenecks, instead computational methods can be effectively applied to search for candidate peptides and identify new promising epitopes.

**Figure 1:**
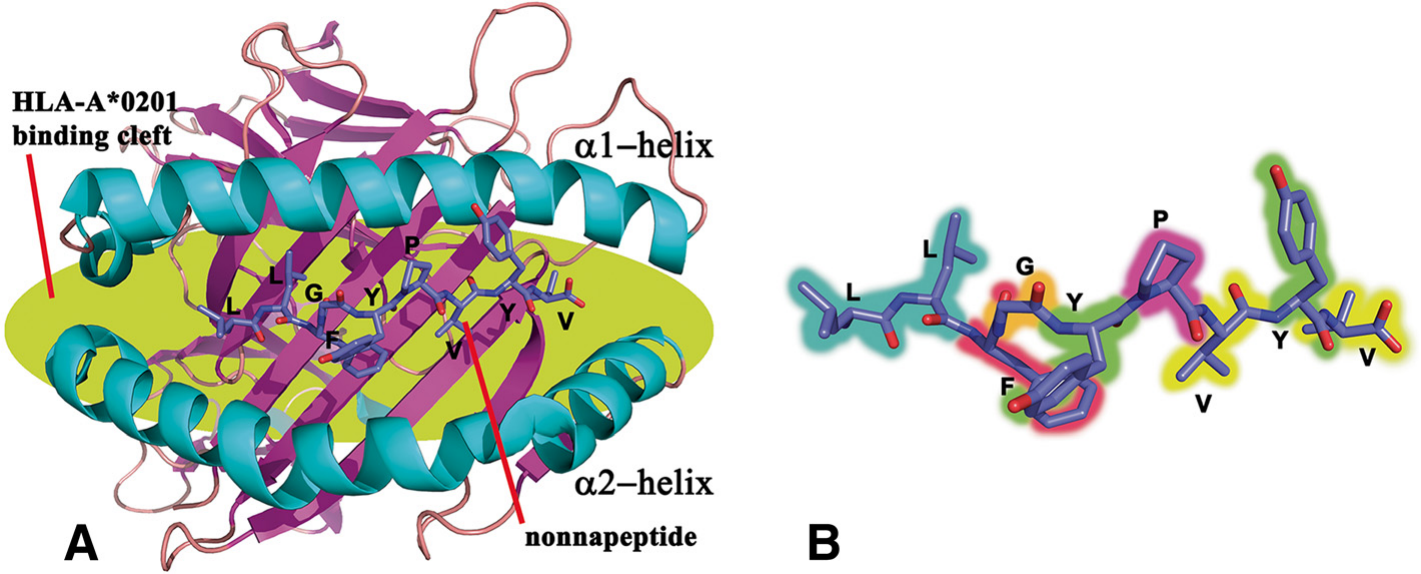
**Visualization of the HLA-nonapeptide complex.** (**A**) Crystal structure of the LLFGYPVYV-HLA-A*02:01 complex resolved by X-ray crystal diffraction (PDB entry 1DUZ [7]) (**B**) Conformation of the nonapeptide extracted from the complex.

Due to the importance of vaccines for human, we focus on MHCs in humans, which are referred to as the human leukocyte antigens (HLAs). There are three classes of HLAs: I, II, and III. Epitopes presented on HLA class I molecules are recognized by CTLs. HLA class I proteins can be categorized into three types according to their genes: HLA-A, HLA-B, and HLA-C. A majority of previous studies have focused on the HLA-A*02:01 allele because it is the most frequent allele of the A2 supertype in the Northeast Asian and Caucasian populations [3]. Typically, the HLA-A*02:01 epitope consists of 8–10 amino acids, and many studies have focused on nonapeptides in particular: that is, epitopes that are 9 residues long [4-6]. Figure 1A shows the nonapeptide epitope LLFGYPVYV fitted inside the HLA-A*02:01 binding cleft, which consists of two α-helices and one β-sheet (from PDB entry 1DUZ [7]). Figure 1B shows the conformation of the nonapeptide epitope LLFGYPVYV.

Early epitope binding prediction algorithms were based on allele-specific motifs [8,9]. For example, for the HLA-A*02:01 allele, positions 2 and 9 of nonapeptides were the most important ones for binding. The residues at both positions were defined as classical anchor residues typically occupied by leucine, valine, and isoleucine since the MHC molecule forms hydrophobic sites for amino acids at these two positions [10]. Additionally, the residues at positions 1, 3, and 7 were identified as secondary anchor residues. Positions 1 and 3 were mainly preferred by tyrosine and phenylalanine [11,12]. The residue at position 7 was suggested to be an amphipathic residue suitable for amino acids with small hydrophobic side-chains such as valine and alanine [13]. In this manner, unknown peptides that matched with such allele-specific motifs were determined to be epitopes.

As more data became available, statistical methods could be applied to calculating a positional scoring matrix. In the matrix, an element was defined individually for each position and specific amino acids, resulting in an *L* × 20 coefficient matrix where *L* is the length of the peptide. In general, the matrix is used under the assumption that each amino acid in a peptide sequence independently contributes to a certain binding energy according to an element included in the positional scoring matrix. Overall binding energy is estimated from the summation of binding energies from all positions. There are several methods based on such a positional scoring matrix: for example, BIMAS [14], RANKPEP [15], Gibbs sampler [16], ARB [17], SMM [18], and SMM^PMBEC^[19].

Currently, the most successful approach for epitope prediction utilizes machine learning algorithms. These algorithms require large enough datasets for training in order to obtain reliable results. Fortunately, the Immune Epitope Database (IEDB) [20] provides more than 100,000 MHC binding data related to T-cell epitopes from infectious pathogens, experimental pathogens, and self-antigens (autoantigens). IEDB encompasses patent data from biotechnological and pharmaceutical companies, as well as direct submissions from research programs and partners. As reliable experimental data are provided, the volume promises a sufficient grounding for developing good predictive models. Although IEDB is not the only database that provides such information, it has more entries than other existing databases. Examples of other databases are SYFPEITHI [21], FIMM [22], MHCPEP [23], MHCBN [24], and AntiJen [25]. NetMHC [26], a predictor based on artificial neural networks, used data from both IEDB and SYFPEITHI and performed very well. SVRMHC [27], a predictor based on support vector regression (SVR) used data from AntiJen and used LIBSVM [28] for SVR-related implementation. Moreover, there also exists an epitope predictor based on a hidden Markov model [29].

The allele-specific motif method, the positional scoring matrix method, and machine learning-based methods use only sequence information in general. Almost none of these methods can provide a clear explanation about the effects of the physicochemical properties of amino acids on binding affinity. In some cases, there are not enough peptides for training: e.g., when using data from rare alleles. Therefore, three-dimensional (3D) structure-based methods have been developed [30-32] to uncover binding mechanisms and address all forces related to binding affinity. However, such methods are currently less reliable than data-driven methods [33]. The reason is that 3D structure-based methods usually require a number of crystal structures of MHC-peptide complexes, which are still not available in large numbers.

Currently, more than 2,000 HLA alleles have been identified. Searching for epitopes that bind to a large number of those alleles would be computationally exhaustive and time-consuming. Therefore, the concept of allele supertypes was developed by clustering alleles into groups based on overlapping epitopes [34-38]. Within each supertype, most of the alleles should share the same epitopes. These epitopes are called ‘promiscuous epitopes’, which show great promise for vaccine development due to their potential for a high level of population coverage.

In this study, we have developed a novel epitope prediction method named EpicCapo. Peptides were encoded numerically by combining information on the peptide-MHC (pMHC) contact sites with amino acid pairwise contact potentials (AAPPs), accompanied by a support vector machine (SVM) [39]. Our method’s performance was evaluated by using benchmark datasets and then compared with other high performance methods. In addition, identification of candidates of promiscuous CTL epitopes for influenza A viruses was demonstrated using the proposed method.

The H1N1 or H5N1 strain of influenza A virus caused a lethal flu in humans, as seen in the epidemics of 2005–2009. Although inactivated influenza vaccination is beneficial, the development of more effective vaccines is still needed, particularly in elderly adults who are more susceptible to viral infections [40]. Identification of promiscuous CTL epitopes might aid this issue by providing candidate peptides from viral proteins for vaccine development.

## Results and discussion

### Comparison of peptide-encoding schemes

**Figure 2:**
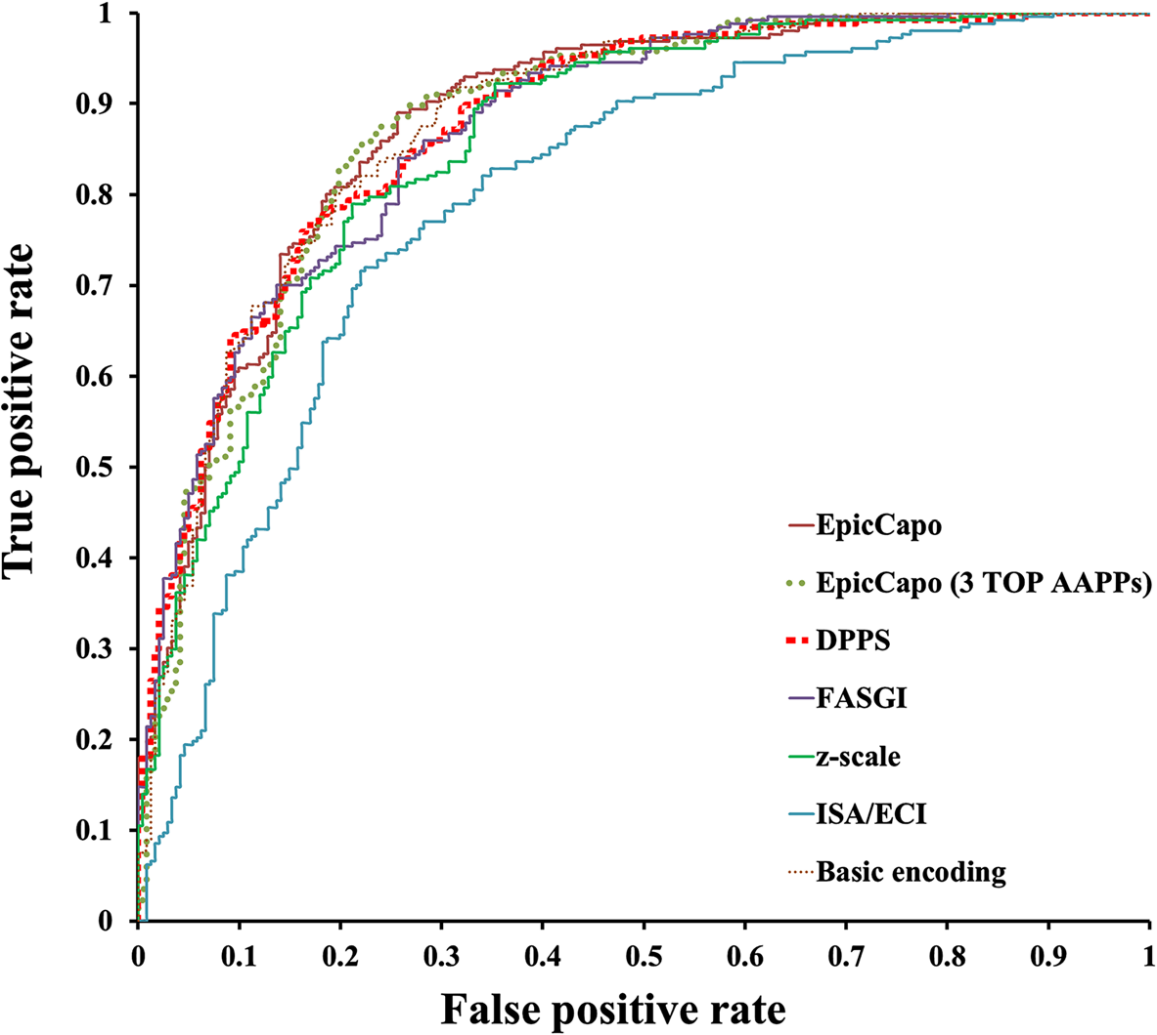
ROC curves of peptide-encoding schemes evaluated on a test set.

We compared our peptide-encoding scheme (Section *Peptide data encoding*) with binary peptide-encoding and with four amino acid descriptors (Table 1). The results of the comparison of the peptide-encoding schemes (Table 2) showed that EpicCapo performed better than others in the classification tasks. It achieved the highest average area under the curve (AUC; 0.882), followed by binary encoding (0.879), DPPS (0.878), FASGAI (0.874), z-scale (0.858), and ISA/ECI (0.796) schemes. All of standard deviations were less than 0.01. A comparison of receiver operating characteristic (ROC) curves is shown in Figure 2.

**Table 1:** Amino acid descriptors acknowledged in this study

**Descriptor**	**Type**	**Technique used**	**# of vector**	**Reference**
DPPS	physicochemical	principal component analysis (PCA)	10	[4]
FASGAI	physicochemical	factor analysis (FA)	6	[41]
z-scale	physicochemical	PCA and partial least square (PLS)	5	[42]
ISA/ECI	quantum-chemical	-	2	[43]

**Table 2:** Classification result of peptide-encoding schemes

**Method**	**# of features**	**10-fold cross validation on training dataset only**					**Holdout method using training dataset and testing dataset**				
		**sens**	**spec**	**F1**	**ACC**	**AUC**	**sens**	**spec**	**F1**	**ACC**	**AUC**
EpicCapo	360	0.883 ± 0.005	0.792 ± 0.006	0.886 ± 0.003	0.841 ± 0.004	0.915 ± 0.001	0.883	0.744	0.831	0.815	0.882
EpicCapo(3 AAPPs*)	27	0.876 ± 0.005	0.821 ± 0.005	0.862 ± 0.003	0.848 ± 0.003	0.916 ± 0.001	0.855	0.777	0.828	0.817	0.878
DPPS	90	0.865 ± 0.005	0.760 ± 0.007	0.834 ± 0.004	0.816 ± 0.004	0.888 ± 0.001	0.868	0.697	0.807	0.785	0.878
FASGAI	54	0.847 ± 0.004	0.761 ± 0.004	0.825 ± 0.003	0.801 ± 0.003	0.882 ± 0.001	0.840	0.730	0.803	0.787	0.874
z-scale	45	0.847 ± 0.005	0.732 ± 0.005	0.815 ± 0.004	0.793 ± 0.004	0.873 ± 0.002	0.848	0.676	0.788	0.765	0.858
ISA/ECI	18	0.799 ± 0.005	0.652 ± 0.005	0.760 ± 0.003	0.731 ± 0.003	0.797 ± 0.001	0.829	0.643	0.766	0.739	0.796
Binary encoding	180	0.883 ± 0.005	0.721 ± 0.006	0.831 ± 0.003	0.807 ± 0.003	0.883 ± 0.002	0.887	0.705	0.820	0.799	0.879

Means and standard deviations were calculated by 20 iterations of 10-fold cross validation.

Underlined values represent the highest performance.

sens = sensitivity; spec = specificity; F1 = F-score; ACC = accuracy; AUC = area under the curve.

*These three top-ranked AAPPs were MICC010101, SIMK990101, and SIMK990105 (see Additional file 1).

Although EpicCapo used the largest number of features (*M* × *K* = 360)—higher than binary encoding (180), DPPS (90), FASGAI (54), z-scale (45), and ISA/ECI (18)—we confirmed that its high performance was not due to a larger number of features. In our study, the training dataset was separated into 40 datasets corresponding to 40 AAPPs. Each dataset consisted of 9 features. The classification functions were fitted to these datasets, and after that the AAPPs were ranked by AUC. The results, as shown in Table 2, suggested that even by using only three top-ranked AAPPs (27 features in total), the classification performance values are comparable to those obtained by using all AAPPs. These three top-ranked AAPPs were MICC010101, SIMK990101, and SIMK990105 (see Additional file 1). They have been previously used in identifying native-like protein structures [44,45], and were also identified as important AAPPs in our accompanying experiments.

### Classification results of benchmark datasets

We applied EpicCapo to benchmark datasets of 34 MHC-I alleles [46]. As shown in Table 3, NetMHC performed the best, ahead of ARB, SMM, and SMM^PMBEC^. For EpicCapo, average AUCs were lower than in NetMHC (0.1%–3.4%) in 13 allele datasets and were higher than in NetMHC (0.1%–9.3%) in 21 allele datasets when using all of the 40 AAPPs (360 features). Almost all of standard deviations were low except several alleles with results of standard deviation larger than 0.01. However, if more data are available, these standard deviations can be decreased. To improve the performance of our method, we developed EpicCapo^+^ by selecting an appropriate subset of AAPPs. As seen in Table 3, the performance of EpicCapo^+^ was higher than EpicCapo and comparable with NetMHC. The overall performance of EpicCapo^+^ is significantly higher than that of other methods according to a paired *t*-test (two-tailed) comparison of average AUCs from all alleles. The IDs of AAPPs used for estimating the predictive models of EpicCapo^+^ are shown in Additional file 2.

**Table 3:** Classification results of 34 allele datasets

**MHC**	**# of peptides**	**AUC**					
		**ARB**	**SMM**	**SMM**^**PMBEC**^	**NetMHC**	**EpicCapo**	**EpicCapo**^**+**^
HLA-A*01:01	1157	0.964	0.980	0.977	0.982	0.972 ± 0.004	0.977 ± 0.003
HLA-A*02:01	3089	0.934	0.952	0.946	0.957	0.950 ± 0.004	0.951 ± 0.004
HLA-A*02:02	1447	0.875	0.899	0.899	0.900	0.901 ± 0.004	**0.909** ± 0.004
HLA-A*02:03	1443	0.884	0.916	0.916	0.921	0.920 ± 0.003	0.923 ± 0.003
HLA-A*02:06	1437	0.872	0.914	0.916	0.927	0.925 ± 0.004	0.927 ± 0.004
HLA-A*03:01	2094	0.908	0.940	0.928	0.937	0.934 ± 0.004	0.938 ± 0.003
HLA-A*11:01	1985	0.918	0.948	0.939	0.951	0.945 ± 0.004	0.951 ± 0.002
HLA-A*24:02	197	0.718	0.780	0.801	0.825	**0.853** ± 0.012	**0.865** ± 0.011
HLA-A*26:01	672	0.907	0.931	0.924	0.956	0.941 ± 0.005	0.957 ± 0.007
HLA-A*29:02	160	0.755	0.911	0.916	0.935	**0.944** ± 0.008	**0.945** ± 0.010
HLA-A*31:01	1869	0.909	0.930	0.925	0.928	0.930 ± 0.002	**0.935** ± 0.003
HLA-A*33:01	1140	0.892	0.925	0.925	0.915	0.926 ± 0.004	**0.934** ± 0.004
HLA-A*68:01	1141	0.840	0.885	0.885	0.883	**0.891** ± 0.003	**0.899** ± 0.003
HLA-A*68:02	1434	0.865	0.898	0.889	0.899	0.901 ± 0.005	0.907 ± 0.003
HLA-B*07:02	1262	0.952	0.964	0.960	0.965	0.960 ± 0.004	0.964 ± 0.002
HLA-B*08:01	708	0.936	0.943	0.956	0.955	0.942 ± 0.005	0.951 ± 0.004
HLA-B*15:01	978	0.900	0.952	0.940	0.941	0.940 ± 0.006	0.950 ± 0.005
HLA-B*18:01	118	0.573	0.853	0.880	0.838	0.886 ± 0.013	**0.911** ± 0.009
HLA-B*27:05	969	0.915	0.940	0.941	0.938	**0.949** ± 0.005	**0.958** ± 0.003
HLA-B*35:01	736	0.851	0.889	0.889	0.875	0.900 ± 0.004	**0.907** ± 0.007
HLA-B*40:02	118	0.541	0.842	0.843	0.754	0.811 ± 0.007	**0.912** ± 0.011
HLA-B*44:02	119	0.533	0.740	0.739	0.778	**0.798** ± 0.009	**0.861** ± 0.013
HLA-B*44:03	119	0.461	0.770	0.753	0.763	**0.813** ± 0.010	**0.871** ± 0.008
HLA-B*51:01	244	0.822	0.868	0.895	0.886	**0.930** ± 0.012	**0.948** ± 0.015
HLA-B*53:01	254	0.871	0.882	0.885	0.899	**0.916** ± 0.008	**0.940** ± 0.008
HLA-B*54:01	255	0.847	0.921	0.935	0.903	0.927 ± 0.008	0.938 ± 0.006
HLA-B*57:01	59	0.428	0.871	0.843	0.826	0.792 ± 0.009	0.854 ± 0.010
HLA-B*58:01	988	0.889	0.964	0.945	0.961	0.959 ± 0.005	0.964 ± 0.004
H-2 Db	303	0.865	0.912	0.901	0.933	**0.940** ± 0.014	**0.968** ± 0.006
H-2 Dd	85	0.696	0.853	0.837	0.925	**0.956** ± 0.016	**0.985** ± 0.017
H-2 Kb	223	0.792	0.810	0.833	0.850	0.844 ± 0.021	**0.880** ± 0.017
H-2 Kd	176	0.798	0.936	0.931	0.939	**0.950** ± 0.015	**0.966** ± 0.009
H-2 Kk	164	0.758	0.770	0.793	0.790	**0.883** ± 0.009	**0.926** ± 0.008
H-2 Ld	102	0.551	0.924	0.942	0.977	**0.984** ± 0.012	**0.992** ± 0.013
Average		0.801	0.895	0.895	0.900	0.912	0.931
*t*-test|ARB		NA	4.37E-5	3.69E-5	1.25E-5	5.21E-6	2.64E-6
*t*-test|SMM			NA	8.61E-1	2.30E-1	8.28E-3	2.87E-5
*t*-test|SMM^PMBEC^				NA	2.61E-1	3.50E-3	8.49E-6
*t*-test|NetMHC					NA	8.57E-3	7.74E-5
*t*-test|EpicCapo						NA	1.95E-5

For each dataset, AUCs were evaluated based on 5-fold cross validation. In the lower part, p-values of average AUCs were calculated using paired *t*-tests (two-tailed).

Means and standard deviations were calculated by 20 iterations of 5-fold cross validation for EpicCapo and EpicCapo^+^.

Underlined values represent the highest performance among ARB, SMM, SMM^PMBEC^, and NetMHC. Values in bold represent significant improvements of EpicCapo or EpicCapo^+^ AUCs from 20 iterations of 5-fold cross validation over the underlined values according to *t*-tests (one-tailed, significance level = 0.01).

### Improved HLA-A-nonapeptide binding predictive models

In this experiment, EpicCapo^+^ was further developed as EpicCapo^+REF^ to improve the predictive performance and identify important positions of nonapeptides in pMHC binding (Section *Improving the performance of HLA-A-nonapeptide binding predictive models*). The IDs of AAPPs used in EpicCapo^+REF^ are shown in Table 4 (for more details on AAPPs, see Additional file 1). The most important AAPPs identified by EpicCapo^+^ were IDs 14 (MICC010101) and 28 (SIMK990105), which were selected in 13 out of 14 alleles. IDs 11 (KESO980102) and 26 (SIMK990103) were also considered to be important, because they were selected in 9 out of 14 alleles. From previous studies that used AAPPs in MHC I epitope prediction, AAPP IDs 19 (MIYS960102) and 2 (BETM990101) proved to be important in peptide-MHC binding predictions [5,47,48]. In our study, however, BETM990101 was not selected for an AAPP subset for any allele, and MIYS960102 was chosen for only two alleles (A*0203 and A*0206). In a report by Schueler-Furman et al. [47], KESO980102 was also tested and compared with MIYS960102; however, there was no significant improvement in the predictive performance. Therefore, it is interesting that MICC010101, SIMK990105, KESO980102, and SIMK990103 were important for generating better predictive models in our study.

**Table 4:** **Optimal subsets of AAPPs and number of selected features identified by EpicCapo**^**+REF **^**using 14 HLA-A allele datasets**

**Allele**	**AUC of EpicCapo**^**+REF**^	**IDs of AAPP used**	**# of features selected**
A *01:01	0.980	1,11,14,20,24,26,28,33	72
A *02:01	0.958	9,11,14,24,26,28,31	62
A *02:02	0.913	14,28	18
A *02:03	0.925	3,9,11,14,19,24,25,26,28,29,31,33	104
A *02:06	0.926	1,3,9,11,13,14,18,19,21,22,24,25,26,27,28,31,34,38,39	141
A *03:01	0.946	11,14,20,24,26,28,33	58
A *11:01	0.956	11,14,26,28	35
A *24:02	0.877	5,6,14,24,28,31	31
A *26:01	0.960	14,28	18
A *29:02	0.955	5,8,9,20,33	23
A *31:01	0.940	11,14,20,26,28,33	46
A *33:01	0.940	14,28	17
A *68:01	0.904	11,14,20,26,28,33	40
A *68:02	0.913	1,9,11,14,20,22,24,26,28,33,39	79
Average	0.935		

We further investigated the generated features according to the selected subset of AAPPs. In our peptide-encoding scheme, nine features were generated from one AAPP, corresponding to the nine amino acid positions in the nonapeptide. Previous studies have indicated that not all positions were important in pMHC binding [4,10-12]. Therefore, some features corresponding to specific positions could be removed to improve the predictive performance.

The Relief algorithm [49] was employed in our study to rank the features according to their importance in separating the nonbinding peptides from the binding ones. The ranking results showed that the ten top-ranked features correspond to positions 9 and 2 in most of the alleles, followed by positions 3, 1, or 7 (see Additional file 3). As indicated in Tables 3 and 4, the overall AUC value of EpicCapo^+REF^ was higher than that of EpicCapo^+^; however, it was still slightly lower than that of NetMHC in the A*01:01 and A*02:06 alleles. In summary, EpicCapo^+REF^ performed better than other methods, with an average AUC of 0.935. Table 4 also shows the number of selected features after employing the Relief-F algorithm. These numbers were different for specific alleles. For the A*01:01, A*02:02, and A*06:01 alleles, no features were removed. However, for the A*02:06, A*24:02, A*29:02, and A*68:02 alleles, 20 or more features were removed. Interestingly, features corresponding to positions 5 and 8, which have previously been considered to not significantly contribute to HLA binding potentials, were still included in some of the selected feature subsets. Therefore, we assumed that features corresponding to different positions are not independent, and that all features from all positions should be required input to estimate the model with the highest-performance (see Additional file 3).

### Candidates of promiscuous epitopes for a development of influenza A viral vaccines

Since EpicCapo^+REF^ performed better than the other existing methods when testing with 14 HLA-A allele datasets, it was further used to find candidates of promiscuous epitopes from influenza A viral sequences. Epitopes from protein sequences of H1N1 (A/PR/8/34), H3N2 (A/Aichi/2/68), H1N1 (A/New York/4290/2009), and H5N1 (A/Hong Kong/483/97) were identified using EpicCapo^+REF^. The prediction results of all influenza A strains categorized into specific alleles are shown in Table 5. All 14 alleles were assigned to supertype groups using the supertype classification defined by previous studies [34-37]. The A*01:01 and A*26:01 alleles were assigned to the A1 group. The A*29:02 allele was assigned to an unidentified group. As shown in Table 5, there are a small number of predicted positive peptides in the A1 supertype. For example, in case of H1N1 (A/PR/8/34), only one peptide was identified as positive for the allele A*26:01. In contrast, there were quite high numbers of predicted positive peptides in the A2, A24, and A3 supertypes. Even the A*29:02 allele, which was assigned to an unidentified group, had a higher number of predicted positive peptides than those in the A1 group. Based on our findings, when promiscuous epitopes were identified from the overlapping epitopes of four Influenza A viral strains (Additional file 4), the A1 group rarely shared peptides with other groups. As shown in Additional file 4, the A*01:01 allele shared only one peptide (YSHGTGTGY) with A*29:02, and the A*26:01 allele shared the peptide DTVNRTHQY with A*29:02 and A*68:01. Moreover, the A*29:02 allele also shared peptides with the A2 and A3 groups: e.g., SMELPSFGV and QTYDWTLNR, respectively (Additional file 4). Therefore, A*29:02 can be considered as a special group that links A1, A2, and A3 together. Furthermore, Doytchinova et al. [38] assigned A*29:02 to the A3 group. However, we did not find overlapping epitopes from the four Influenza A viral strains in the A*24:02 allele assigned to the A24 group. This suggested that A*24:02 itself is different from other alleles considered here, and this might be the reason why most of the previous studies assigned it separately to the A24 group [34-37]. As shown in Additional file 4, 51 peptides (67.1%) of the total 76 epitopes were immunologically validated as positive, whereas 9 peptides (11.8%) were validated as negative. No evidence of immunological validation could be obtained for 16 peptides (21.1%). These results indicate that our newly developed method provides a markedly high accuracy in epitope identification, given the fact that most of the identified epitopes could be correlated with immunological experimental evidence. However, even without such immunological evidence, those epitopes identified by our computational approach might be considered as candidates for new vaccine development.

**Table 5:** **Prediction results of EpicCapo**^**+REF **^**using four influenza A strains categorized by specific alleles**

**Allele**	**# of predicted positive peptides**				**Super type**
	**H1N1 New York/4290/2009**	**H5N1 Hong Kong/483/97**	**H1N1 PR/8/34**	**H3N2 Aichi/2/68**	
A *01:01	14	13	6	5	A1
A *26:01	6	9	1	5	A1
A *29:02	103	134	61	161	?
A *02:01	122	160	71	168	A2
A *02:02	302	370	162	391	A2
A *02:03	268	326	144	307	A2
A *02:06	200	250	105	264	A2
A *68:02	198	220	109	277	A2
A *24:02	90	108	50	150	A24
A *03:01	85	94	50	136	A3
A *11:01	162	176	91	229	A3
A *31:01	183	227	110	245	A3
A *33:01	96	117	62	110	A3
A *68:01	263	346	151	325	A3
Total	2092	2550	1173	2773	

Our results are in agreement with the study by Uchida [50], which identified promiscuous epitopes from influenza A H1N1 (A/PR/8/34), H3N2 (A/Aichi/2/68), H1N1 (A/New York/4290/2009), and H5N1 (A/Hong Kong/483/97). Uchida found experimentally confirmed CTL epitopes in the A2 group. In our results, the epitopes identified by EpicCapo^+REF^ in the A2 group were consistent with them (Table 6). In addition, we found promising candidates of promiscuous epitopes also for the A1 and A3 groups as shown in Additional file 4.

**Table 6:** **Comparison of epitopes identified by EpicCapo**^**+REF **^**with the broadly protective influenza A viral epitopes identified by Uchida**[50]

**Viral strain**	**CTL epitopes identified by[**[50]**]**	**Shared alleles identified by EpicCapo**^**+REF**^
H1N1 (A/PR/8/34)	GILGFVFTL	A*02:01, A*02:02, A*02:03, A*02:06
	IILKANFSV	A*02:01, A*02:02, A*02:03, A*02:06, A*68:02
	GMFNMLSTV	A*02:01, A*02:02, A*02:03, A*02:06
H3N2 (A/Aichi/2/68)	GILGFVFTL	A*02:01, A*02:02, A*02:03, A*02:06
	VMLKANFSV	A*02:01, A*02:02, A*02:03, A*02:06
	GMFNMLSTV	A*02:01, A*02:02, A*02:03, A*02:06
H1N1 (A/NewYork/4290/2009)	GILGFVFTL	A*02:01, A*02:02, A*02:03, A*02:06
	IVLKANFSV	A*02:01, A*02:02, A*02:06, A*68:02
	GMFNMLSTV	A*02:01, A*02:02, A*02:03, A*02:06
H5N1 (A/Hong Kong/483/97)	GILGFVFTL	A*02:01, A*02:02, A*02:03, A*02:06
	IILKANFSV	A*02:01, A*02:02, A*02:03, A*02:06, A*68:02
	GMFNMLSTV	A*02:01, A*02:02, A*02:03, A*02:06

Although the overall performance of EpicCapo^+REF^ was high, there are two limitations in the use of this method. The first limitation is the length of input peptides must be equal to 9. In the further study, we will improve EpicCapo^+REF^ to be applicable to peptides with the length of 8–11. The second limitation is that input amino acids must not be special or ambiguous ones. Examples of special amino acids are U (Selenocysteine) and O (Pyrrolysine). Also, examples of ambiguous amino acids are B (Asparagine or aspartic acid), Z (Glutamine or glutamic acid), and J (Leucine or Isoleucine). EpicCapo^+REF^ are not applicable with these amino acids since they are not included in AAPPs.

## Conclusions

In this study, we have developed a novel method for epitope prediction. Peptides were encoded numerically, combining information of pMHC contact sites and amino acid pairwise contact potentials, accompanied by an SVM for estimating the predictive model. Our method achieved high performance in testing with benchmark datasets. In addition, our study identified a number of candidates of promiscuous CTL epitopes from four influenza A viral strains, consistent with previously reported immunological experiments. This consistency in results strongly supports the accuracy of our method. We speculate that our techniques may be useful in identifying promising candidates of promiscuous epitopes for the development of new vaccines.

## Methods

### Peptide data encoding

We propose a novel peptide-encoding scheme for machine learning algorithms. This scheme utilized the information of pMHC contact sites retrieved from the international ImMunoGeneTics information system, IMGT [51], the allele-specific positional scoring matrices developed by SMM^PMBEC^[19], and the AAPPs from AAindex [52].

The reference pMHC contact sites retrieved from IMGT were modified by adding more MHC positions. The added MHC positions were determined by observing the pMHC contact sites of the selected 189 crystal structures of the HLA-nonapeptide complex collected from IMGT entries specific to the MHC-I receptor type. If there were new contact positions, the reference pMHC contact sites were modified by adding those new positions. Therefore, more HLA-nonapeptide contact positions were included in the modified pMHC contact site because the reference pMHC contact sites resulted from the use of only 74 crystal structures of the HLA-nonapeptide complex [51]. Utilizing the modified pMHC contact sites should provide more reliable results during the prediction. Additional file 5 shows the references and added pMHC contact sites positions. This information served as a binding template between the peptide and MHC. In NetMHCpan [53], the reference pMHC contact sites were used to extract a pseudosequence representing the given MHC molecule. When performing prediction, sequence information from both peptide and MHC was taken into account. However, the pairs of amino acids between the MHC molecule and peptide were not of concern. Therefore, to generate a more informative predictive model, we used information about the pairs of amino acids at the interface between an MHC molecule and a nonapeptide, represented by AAPPs. In addition, the allele-specific positional scoring matrices developed by SMM^PMBEC^ were used in our study. These matrices provide information of how likely a given amino acid would be preferred or avoided in a specific residue. Like NetMHCpan, SMM^PMBEC^ did not use AAPPs. Consequently, we proved that a proper selection of AAPPs could lead to higher performance in the prediction. The encoded data could be further used in tasks of classification or regression using machine learning algorithms. In this study, we demonstrated the feasibility of the classification task by using the SVM implemented in the R package kernlab [39].

**Figure 3:**
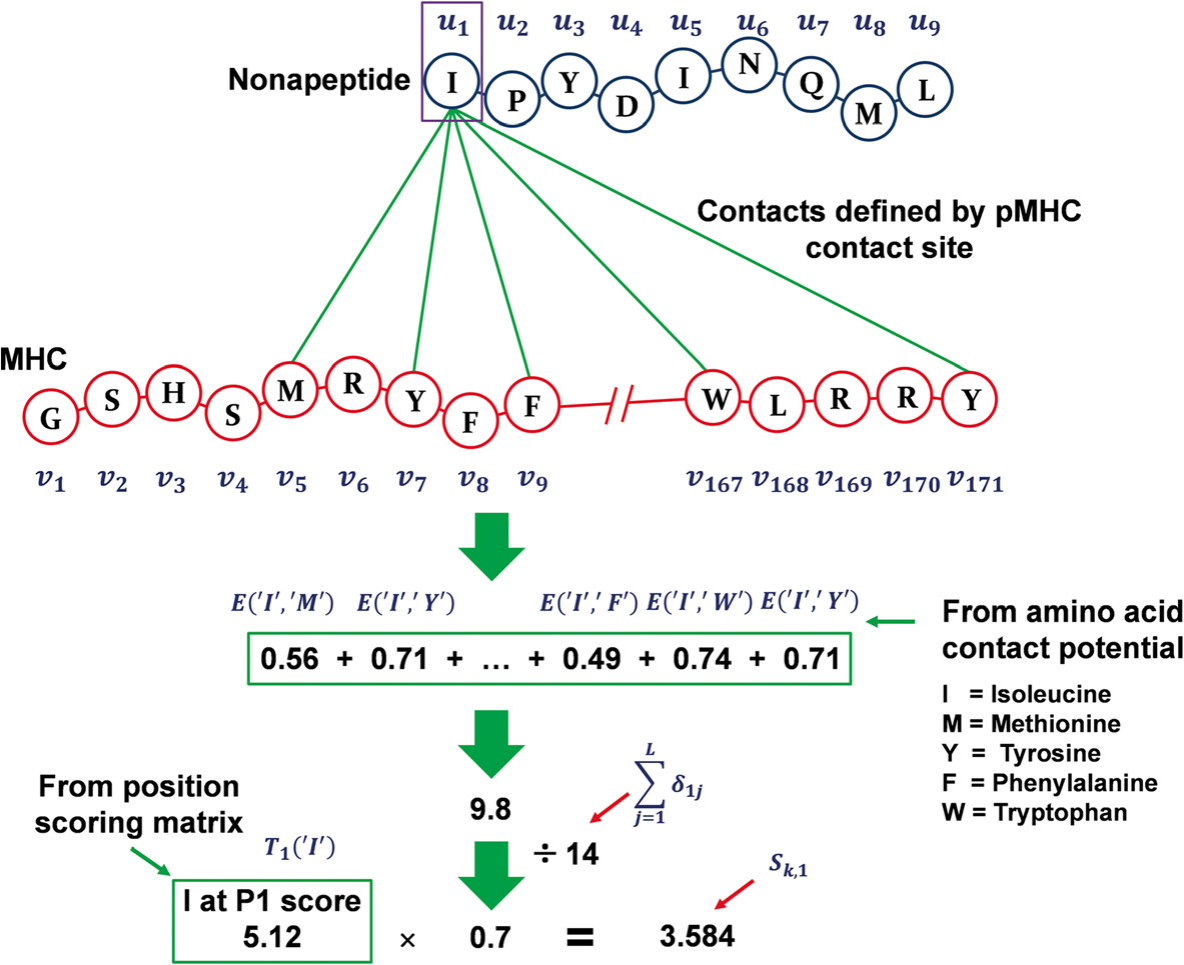
Our peptide data-encoding scheme, using the first position of a nonapeptide as an example.

Here, we propose a novel scheme for encoding nonapeptides into input vectors of the SVM. Suppose *E*(*a*_1_,*a*_2_) is an AAPP for the amino acids *a*_1_ and *a*_2_. If two or more types of AAPPs are available, we denote *k*th type of the AAPP by *E*_*k*_(*a*_1_,*a*_2_). Also, we denote the *i*th amino acid of the nonapeptide *n* and the *j*th amino acid of HLA by *u*_*i*_^(*n*)^ and *v*_*j*_, respectively. In order to combine information of position-specific amino acid scores of the nonapeptides with AAPPs, we define a score *S*_*k*,*i*_^(*n*)^ for the *i*th a *k*th type of AAPP as follows:

$$S_{k,i}^{\left(n\right)}=T_{i}\left(u_{i}^{\left(n\right)}\right)\cdot \left(\sum_{j=1}^{L}\delta_{\mathrm{ij}}E_{k}\left(u_{i}^{\left(n\right)},v_{j}\right)/\sum_{j=1}^{L}\delta_{\mathrm{ij}}\right)\text{,}$$

where *L* is the length of the HLA protein, *T*_*i*_(*a*) is the *i*th position score of the amino acid *a* for the nonapeptides described by SMM^PMBEC^, and *δ*_*ij*_ is an indicator variable that takes the value of 1 if the *i*th amino acid of a nonapeptide and the *j*th amino acid of HLA contact each other, and 0 otherwise. Here, the positional scoring matrix *T*_*i*_(*a*) is trained based on training data and multiplied by −1 to reverse the order of values (a high positive value denotes high preference between an amino acid and the position) and scaled into the range of 1 to 10 since we need to avoid loss of information when *T*_*i*_(*a*) equals zero. In fact, any range that does not include zero can be used; in this study, it is the range of 1 to 10. The scaling of positional scoring matrices is shown in Additional file 6. Note that ∑ _*j* = 1_^*L*^*δ*_*ij*_ is the number of contact sites for the *i*th amino acid of a nonapeptide (see Additional file 5). Intuitively, this score represents average pair-potential of contact sites, weighted by position-specific amino acid score for nonapeptides. Let *K* be the number of AAPPs available, and *M* be the length of the peptide, set to 9 throughout this study. Using this scoring scheme, we transform a nonapeptide *n* into a *M* × *K*-dimensional numerical vector, whose (*M*(*k*–1) + *i*)^th^ element is *S*_*k*,*i*_^(*n*)^. For example, the encoded nonapeptides consist of 9 features if one AAPP is used, and 360 features if 40 AAPPs are used. Figure 3 illustrates an example of the data-encoding scheme for the first position of the nonapeptide.

Our peptide-encoding scheme was compared with binary peptide-encoding and with four amino acid descriptors, as shown in Table 1 using the dataset reported by Bi and colleagues (supplementary information for Table S2 in [54]). This dataset consists of 1,998 quantitative affinity-known HLA-A*02:01-restricted nonapeptides. The dataset was randomly partitioned into a training set containing 1,500 nonapeptides for estimating predictive models using the SVM, and a test set containing 498 nonapeptides for validating the models. For our peptide-encoding scheme, the positional scoring matrix was trained based on the external dataset downloaded from IEDB, consisting of 500 nonapeptides restricted to the HLA-A*02:01 allele (Additional file 7). These nonapeptides were included in neither training nor test sets. For the binary peptide-encoding, each amino acid was encoded as a binary vector of length 20, resulting in a vector of length 180 for a nonapeptide. In case of using amino acid descriptors, the length of an encoded vector would be equal to *M* times larger than the length of descriptor vectors. The performances of the data-encoding schemes were evaluated in classification tasks, using a 10-fold cross validation. Throughout our experiments, the parameter C (cost of constraint violation), epsilon, and the type of kernel used for the SVM were 1, 0.1, and the radial basis kernel, respectively. The class for each nonapeptide was determined by using an IC_50_ affinity cutoff at 500 nM. Nonapeptides with an affinity less than 500 nM were considered to be binders, and non-binders otherwise. The study by Moutaftsi et al. [55] showed that 90 of epitopes that could stimulate CD8^+^ T cell responses bound to MHC with affinities lower than 500 nM. The predictive performance is evaluated using five measures: overall accuracy (ACC), sensitivity (sens), specificity (spec), F-score (F1), and area under the curve (AUC) for the received operating characteristic curve. ACC, sens, spec, and F1 are defined as

$$\text{ACC}=\frac{\text{TP}+\text{TN}}{\text{TP}+\text{TN}+\text{FP}+\text{FN}}\text{,}$$

$$\text{sens}=\frac{\text{TP}}{\text{TP}+\text{FN}}\text{,}$$

$$\text{spec}=\frac{\text{TN}}{\text{FP}+\text{TN}}\text{,}$$

$$\text{F}1=\frac{2\times \text{TP}}{\left(\left(2\times \text{TP}\right)+\text{FN}+\text{FP}\right)}\text{,}$$

where TP, FP, TN, and FN are the numbers of overall true positives, false positives, true negatives, and false negatives, respectively.

### Validation of predictive models using benchmark datasets

The performance of EpicCapo was validated by using benchmark datasets of 34 MHC-I alleles provided by Peters et al. [46]. In this experiment, the positional scoring matrices were trained based on training data according to the cross validation technique. 20 iterations of 5-fold cross validation were conducted to evaluate AUCs for EpicCapo. We compared the results of our method with those of ARB, NetMHC, SMM, and SMM^PMBEC^.

EpicCapo was further developed as EpicCapo^+^ by selecting AAPPs. Each encoded allele dataset was initially separated into 40 datasets according to the 40 AAPPs. The classification task was performed for each dataset to calculate AUC using the SVM and using the same parameters as EpicCapo. Then, the 40 datasets were ranked by AUC from highest to lowest. Next, the classification task was performed again by adding the datasets of AAPPs one by one based on their rank. Finally, the optimal subset of AAPPs that led to the highest AUC was identified for each allele. The average AUCs of all alleles as calculated from EpicCapo^+^ were compared with those from EpicCapo and other methods using paired *t*-tests (two-tailed). For each allele, the AUCs from 20 iterations of 5-fold cross validation of EpicCapo and EpicCapo^+^ were compared with the maximum AUC among other methods by using *t*-tests (one-tailed, significance level = 0.01).

### Improving the performance of HLA-A-nonapeptide binding predictive models

To increase the performance of our predictive models, the positional scoring matrices used in this experiment were trained based on datasets containing larger number of nonapeptides. These matrices are available at [56]. After encoding 14 HLA-A allele datasets using the downloaded matrices, EpicCapo^+^ was performed again to identify optimal subsets of AAPPs therein. We used the Relief-F algorithm [49] implemented in the machine learning software Weka [57] to perform the feature selection task, ranking the features according to their importance in discriminating the MHC binder peptides from the non-binder ones. The default parameters provided by Weka were used, and a 5-fold cross validation was conducted for evaluating feature importance. The best feature subsets were constructed by adding the features, one by one, from the top-ranked feature to the last one in the classification task using the SVM. The AUC gradually increased with the addition of features, until it reached the highest value. Features after this point were considered irrelevant and ignored. We named this method, accompanied with the Relief-F algorithm, EpicCapo^+REF^.

### Identification of candidates of promiscuous epitopes

EpicCapo^+REF^ was further tested to identify candidates of promiscuous epitopes—i.e., nonapeptides that were predicted to be MHC binders for various HLA alleles—from the protein sequences of four influenza A viral subtypes: H1N1 (A/PR/8/34), H3N2 (A/Aichi/2/68), H1N1 (A/New York/4290/2009), and H5N1 (A/Hong Kong/483/97). These protein sequences were downloaded from the NCBI website (http://www.ncbi.nlm.nih.gov/). The nonapeptides were generated from these sequences by using a nonamer sliding window. Next, all of the generated nonapeptides were used as inputs in EpicCapo^+REF^ predictive models. These models were estimated by using 14 HLA-A allele datasets, and each model was specific for each allele type. The identified epitopes were validated by cross-checking with the results of immunological experiments.

## Competing interests

The authors declare that they have no competing interests.

## Authors’ contributions

TS and KS defined the research question, and designed and performed the experiments. OH, IK, YY, and MK drafted the manuscript. All authors contributed to and approved the final version of the manuscript.

## Supplementary Material

Additional file 1 Amino acid pairwise contact potentials (AAPPs) used in this study (http://www.genome.jp/aaindex/). Click here for file

Additional file 2 **Optimal subsets of AAPPs identified by EpicCapo**^**+**^**using 34 benchmark datasets.** Click here for file

Additional file 3 **Features selected by EpicCapo**^**+REF **^**separated in each allele.** The rank indicates importance of feature. Click here for file

Additional file 4 Candidates of promiscuous epitopes identified from overlapping epitopes of influenza A viral strains: H1N1 (A/New York/4290/2009), H5N1 (A/Hong Kong/483/97), H1N1 (A/PR/8/34), and H3N2 (A/Aichi/2/68). Click here for file

Additional file 5 Reference and added pMHC contact sites for HLA. Click here for file

Additional file 6 The scaling of positional scoring matrices. Click here for file

Additional file 7 The positional scoring matrix of EpicCapo used in the experiment that compared peptide-encoding schemes. Click here for file
