# Supplementary material for: EpicCapo: epitope prediction using combined information of amino acid pairwise contact potentials and HLA-peptide contact site information
Source: BMC Bioinformatics. 2012 Nov 24;13:313. doi: 10.1186/1471-2105-13-313 (PMC3548761; doi:10.1186/1471-2105-13-313)
Supplement: Additional file 1 — Amino acid pairwise contact potentials (AAPPs) used in this study (http://www.genome.jp/aaindex/). [file 1471-2105-13-313-S1.doc]

**Additional file 1 - Amino acid pairwise contact potentials (AAPPs) used in this study (retrieved from [49],** [**http://www.genome.jp/aaindex/**](http://www.genome.jp/aaindex/)**).**

| **ID** | **Accession #** | **Description** | **Ref.** |
| --- | --- | --- | --- |
| **1** | BASU010101 | Optimization-based potential derived by the modified perceptron criterion | [1] |
| **2** | BETM990101 | Modified version of the Miyazawa-Jernigan transfer energy | [2] |
| **3** | BONM030101 | Quasichemical statistical potential for the antiparallel orientation of interacting side groups | [3] |
| **4** | BONM030102 | Quasichemical statistical potential for the intermediate orientation of interacting side groups | [3] |
| **5** | BONM030103 | Quasichemical statistical potential for the parallel orientation of interacting side groups | [3] |
| **6** | BONM030104 | Distances between centers of interacting side chains in the antiparallel orientation | [3] |
| **7** | BONM030105 | Distances between centers of interacting side chains in the intermediate orientation | [3] |
| **8** | BONM030106 | Distances between centers of interacting side chains in the parallel orientation | [3] |
| **9** | BRYS930101 | Distance-dependent statistical potential (only energies of contacts within 0–5 Angstroms are included) | [4] |
| **10** | KESO980101 | Quasichemical transfer energy derived from interfacial regions of protein-protein complexes | [5] |
| **11** | KESO980102 | Quasichemical energy in an average protein environment derived from interfacial regions of protein-protein complexes | [5] |
| **12** | KOLA930101 | Statistical potential derived by the quasichemical approximation | [6] |
| **13** | LIWA970101 | Modified version of the Miyazawa-Jernigan transfer energy | [7] |
| **14** | MICC010101 | Optimization-derived potential | [8] |
| **15** | MIRL960101 | Statistical potential derived by the maximization of the harmonic mean of Z scores | [9] |
| **16** | MIYS850102 | Quasichemical energy of transfer of amino acids from water to the protein environment | [10] |
| **17** | MIYS850103 | Quasichemical energy of interactions in an average buried environment | [10] |
| **18** | MIYS960101 | Quasichemical energy of transfer of amino acids from water to the protein environment | [11] |
| **19** | MIYS960102 | Quasichemical energy of interactions in an average buried environment | [11] |
| **20** | MIYS960103 | Number of contacts between side chains derived from 1168 X-ray protein structures | [11] |
| **21** | MIYS990106 | Quasichemical energy of transfer of amino acids from water to the protein environment | [12] |
| **22** | MIYS990107 | Quasichemical energy of interactions in an average buried environment | [12] |
| **23** | MOOG990101 | Quasichemical potential derived from interfacial regions of protein-protein complexes | [13] |
| **24** | SIMK990101 | Distance-dependent statistical potential (contacts within 0–5 Angstroms) | [14] |
| **25** | SIMK990102 | Distance-dependent statistical potential (contacts within 5–7.5 Angstroms) | [14] |
| **26** | SIMK990103 | Distance-dependent statistical potential (contacts within 7.5–10 Angstroms) | [14] |
| **27** | SIMK990104 | Distance-dependent statistical potential (contacts within 10–12 Angstroms) | [14] |
| **28** | SIMK990105 | Distance-dependent statistical potential (contacts longer than 12 Angstroms) | [14] |
| **29** | SKOJ000101 | Statistical quasichemical potential with the partially composition-corrected pair scale | [15] |
| **30** | SKOJ000102 | Statistical quasichemical potential with the composition-corrected pair scale | [15] |
| **31** | SKOJ970101 | Statistical potential derived by the quasichemical approximation | [16] |
| **32** | TANS760101 | Statistical contact potential derived from 25 X-ray protein structures | [17] |
| **33** | TANS760102 | Number of contacts between side chains derived from 25 X-ray protein structures | [17] |
| **34** | THOP960101 | Mixed quasichemical and optimization-based protein contact potential | [18] |
| **35** | TOBD000101 | Optimization-derived potential obtained for small set of decoys | [19] |
| **36** | TOBD000102 | Optimization-derived potential obtained for large set of decoys | [19] |
| **37** | VENM980101 | Statistical potential derived by the maximization of the perceptron criterion | [20] |
| **38** | ZHAC000101 | Environment-dependent residue contact energies (rows = helix, cols = helix) | [21] |
| **39** | ZHAC000104 | Environment-dependent residue contact energies (rows = strand, cols = strand) | [21] |
| **40** | ZHAC000106 | Environment-dependent residue contact energies (rows = coil, cols = coil) | [21] |

**References**

1. Bastolla U, Farwer J, Knapp EW, Vendruscolo M: **How to guarantee optimal stability for most representative structures in the Protein Data Bank.** *Proteins* 2001, **44:**79-96.

2. Betancourt MR, Thirumalai D: **Pair potentials for protein folding: choice of reference states and sensitivity of predicted native states to variations in the interaction schemes.** *Protein Sci* 1999, **8:**361-369.

3. Boniecki M, Rotkiewicz P, Skolnick J, Kolinski A: **Protein fragment reconstruction using various modeling techniques.** *J Comput Aided Mol Des* 2003, **17:**725-738.

4. Bryant SH, Lawrence CE: **An empirical energy function for threading protein sequence through the folding motif.** *Proteins* 1993, **16:**92-112.

5. Keskin O, Bahar I, Badretdinov AY, Ptitsyn OB, Jernigan RL: **Empirical solvent-mediated potentials hold for both intra-molecular and inter-molecular inter-residue interactions.** *Protein Sci* 1998, **7:**2578-2586.

6. Kolinski A, Godzik A, Skolnick J: **A General-Method for the Prediction of the 3-Dimensional Structure and Folding Pathway of Globular-Proteins - Application to Designed Helical Proteins.** *J Chem Phys* 1993, **98:**7420-7433.

7. Liwo A, Oldziej S, Pincus MR, Wawak RJ, Rackovsky S, Scheraga HA: **A united-residue force field for off-lattice protein-structure simulations .1. Functional forms and parameters of long-range side-chain interaction potentials from protein crystal data.** *J Comput Chem* 1997, **18:**849-873.

8. Micheletti C, Seno F, Banavar JR, Maritan A: **Learning effective amino acid interactions through iterative stochastic techniques.** *Proteins* 2001, **42:**422-431.

9. Mirny LA, Shakhnovich EI: **How to derive a protein folding potential? A new approach to an old problem.** *Journal of Molecular Biology* 1996, **264:**1164-1179.

10. Miyazawa S, Jernigan RL: **Estimation of Effective Interresidue Contact Energies from Protein Crystal-Structures-Quasi-Chemical Approximation.** *J Macromolecules* 1985, **18:**534-552.

11. Miyazawa S, Jernigan RL: **Residue-residue potentials with a favorable contact pair term and an unfavorable high packing density term, for simulation and threading.** *Journal of Molecular Biology* 1996, **256:**623-644.

12. Miyazawa S, Jernigan RL: **Self-consistent estimation of inter-residue protein contact energies based on an equilibrium mixture approximation of residues.** *Proteins* 1999, **34:**49-68.

13. Moont G, Gabb HA, Sternberg MJE: **Use of pair potentials across protein interfaces in screening predicted docked complexes.** *Proteins-Structure Function and Genetics* 1999, **35:**364-373.

14. Simons KT, Ruczinski I, Kooperberg C, Fox BA, Bystroff C, Baker D: **Improved recognition of native-like protein structures using a combination of sequence-dependent and sequence-independent features of proteins.** *Proteins* 1999, **34:**82-95.

15. Skolnick J, Jaroszewski L, Kolinski A, Godzik A: **Derivation and testing of pair potentials for protein folding. When is the quasichemical approximation correct?** *Protein Sci* 1997, **6:**676-688.

16. Skolnick J, Kolinski A, Ortiz A: **Derivation of protein-specific pair potentials based on weak sequence fragment similarity.** *Proteins* 2000, **38:**3-16.

17. Tanaka S, Scheraga HA: **Medium- and long-range interaction parameters between amino acids for predicting three-dimensional structures of proteins.** *Macromolecules* 1976, **9:**945-950.

18. Thomas PD, Dill KA: **An iterative method for extracting energy-like quantities from protein structures.** *Proc Natl Acad Sci U S A* 1996, **93:**11628-11633.

19. Tobi D, Shafran G, Linial N, Elber R: **On the design and analysis of protein folding potentials.** *Proteins* 2000, **40:**71-85.

20. Vendruscolo M, Domany E: **Pairwise contact potentials are unsuitable for protein folding.** *J Chem Phys* 1998, **109:**11101-11108.

21. Zhang C, Kim SH: **Environment-dependent residue contact energies for proteins.** *P Natl Acad Sci USA* 2000, **97:**2550-2555.
