# Supplementary material for: EpicCapo: epitope prediction using combined information of amino acid pairwise contact potentials and HLA-peptide contact site information
Source: BMC Bioinformatics. 2012 Nov 24;13:313. doi: 10.1186/1471-2105-13-313 (PMC3548761; doi:10.1186/1471-2105-13-313)
Supplement: Additional file 2 — Optimal subsets of AAPPs identified by EpicCapo+using 34 benchmark datasets. [file 1471-2105-13-313-S2.doc]

**Additional file 2 - Optimal subsets of AAPPs identified by EpicCapo^+^ using 34 benchmark datasets.**

| **MHC** | **IDs of AAPP used** |
| --- | --- |
| HLA-A*0101 | 11,14,20,24,28,33 |
| HLA-A*0201 | 9,11,14,24,26,28,31 |
| HLA-A*0202 | 14,24,28 |
| HLA-A*0203 | 3,9,11,14,19,24,25,26,28,29,31 |
| HLA-A*0206 | 9,11,13,14,19,21,22,24,25,26,28,31 |
| HLA-A*0301 | 9,11,14,20,24,26,28,33 |
| HLA-A*1101 | 11,14,26,28 |
| HLA-A*2402 | 11,14,20,24,28,31,33 |
| HLA-A*2601 | 14,28 |
| HLA-A*2902 | 5,9,11,14,19,20,22,24,26,28,33 |
| HLA-A*3101 | 1,9,11,14,20,24,26,28,31,33,38 |
| HLA-A*3301 | 1,11,14,20,24,26,28,33 |
| HLA-A*6801 | 11,14,20,26,28 |
| HLA-A*6802 | 1,2,9,11,14,19,20,22,24,26,28,33,34,39 |
| HLA-B*0702 | 1,9,11,14,20,24,26,28,33 |
| HLA-B*0801 | 4,14,18,20,40 |
| HLA-B*1501 | 14,24,26,28 |
| HLA-B*1801 | 3,14,20,24,26,28 |
| HLA-B*2705 | 9,14,20 |
| HLA-B*3501 | 14,28 |
| HLA-B*4002 | 11,14,24,28 |
| HLA-B*4402 | 9,14,20,28,32 |
| HLA-B*4403 | 13,14,20,28,33,38,39 |
| HLA-B*5101 | 6,11,14,20,24,26,33,36,38,39 |
| HLA-B*5301 | 11,14,20,24,28,33 |
| HLA-B*5401 | 1,9,11,14,20,24,26,28,33 |
| HLA-B*5701 | 5,6,8,12,22,23,24,25,27,31,37 |
| HLA-B*5801 | 14,28 |
| H-2 Db | 1,11,14,24,28 |
| H-2 Dd | 11,14,28 |
| H-2 Kb | 11,14,28 |
| H-2 Kd | 1,11,12,14,19,24,26,28,33 |
| H-2 Kk | 14,28 |
| H-2 Ld | 10,11,14,16,18,20,21,23,24,26,28,33 |
