# Supplementary material for: EpicCapo: epitope prediction using combined information of amino acid pairwise contact potentials and HLA-peptide contact site information
Source: BMC Bioinformatics. 2012 Nov 24;13:313. doi: 10.1186/1471-2105-13-313 (PMC3548761; doi:10.1186/1471-2105-13-313)
Supplement: Additional file 4 — Candidates of promiscuous epitopes identified from overlapping epitopes of influenza A viral strains: H1N1 (A/New York/4290/2009), H5N1 (A/Hong Kong/483/97), H1N1 (A/PR/8/34), and H3N2 (A/Aichi/2/68). [file 1471-2105-13-313-S4.doc]

## Additional file 4 - Candidates of promiscuous epitopes identified from overlapping epitopes of influenza A viral strains: H1N1 (A/New York/4290/2009), H5N1 (A/Hong Kong/483/97), H1N1 (A/PR/8/34), and H3N2 (A/Aichi/2/68).

| **Epitope** | **Shared alleles** | **Ref.** | **T cell assay** |
| --- | --- | --- | --- |
| **QTYDWTLNR** | A*0301, A*1101, A*2902, A*3101, A*3301, A*6801 | [1] | Positive |
| **KFFPSSSYR** | A*0301, A*1101, A*2902, A*3101, A*3301, A*6801 | [1] | Positive |
| **MMMGMFNML** | A*0201, A*0202, A*0203, A*0206, A*6802 | [2] | Positive |
| **FVANFSMEL** | A*0201, A*0202, A*0203, A*0206, A*6802 | [2] | Positive |
| **LLTEVETYV** | A*0201, A*0202, A*0203, A*0206, A*6802 | [3] | Positive |
| **ALASCMGLI** | A*0201, A*0202, A*0203, A*0206, A*6802 | [4] | Negative |
| **IMFSNKMAR** | A*0301, A*1101, A*3101, A*3301, A*6801 | [5] | Positive |
| **RLFFKCIYR** | A*0301, A*1101, A*3101, A*3301, A*6801 | [1] | Positive |
| **AQTDCVLEA** | A*0201, A*0202, A*0203, A*0206 | - | - |
| **RLIDFLKDV** | A*0201, A*0202, A*0203, A*0206 | [6] | Positive |
| **GMFNMLSTV** | A*0201, A*0202, A*0203, A*0206 | [7] | Positive |
| **NMLSTVLGV** | A*0201, A*0202, A*0203, A*0206 | [8] | Positive |
| **AQMALQLFI** | A*0201, A*0202, A*0203, A*0206 | - | - |
| **KICSTIEEL** | A*0201, A*0202, A*0203, A*0206 | [5] | Positive |
| **AIVGEISPL** | A*0201, A*0202, A*0203, A*0206 | [5] | Positive |
| **GILGFVFTL** | A*0201, A*0202, A*0203, A*0206 | [9] | Positive |
| **SMELPSFGV** | A*0201, A*0202, A*0203, A*2902 | [1] | Positive |
| **GMMMGMFNM** | A*0201, A*0203, A*0206, A*2902 | [16] | Negative |
| **CVLEAMAFL** | A*0201, A*0203, A*0206, A*6802 | [1] | Negative |
| **MINNDLGPA** | A*0202, A*0203, A*0206, A*6802 | [10] | Positive |
| **YGFVANFSM** | A*0202, A*0206, A*2902, A*6802 | [16] | Positive |
| **MSIGVTVIK** | A*0301, A*1101, A*3101, A*6801 | [5] | Positive |
| **ATTHSWIPK** | A*0301, A*1101, A*3101, A*6801 | [1] | Positive |
| **MVLASTTAK** | A*0301, A*1101, A*3101, A*6801 | [6] | Positive |
| **FEFTSFFYR** | A*0301, A*2902, A*3101, A*6801 | [5] | Positive |
| **LANTIEVFR** | A*1101, A*3101, A*3301, A*6801 | - | - |
| **NTMTKDAER** | A*1101, A*3101, A*3301, A*6801 | [15] | Positive |
| **TTHSWIPKR** | A*1101, A*3101, A*3301, A*6801 | [15] | Positive |
| **KLANVVRKM** | A*0201, A*0202, A*0203 | [16] | Positive |
| **VLGVSILNL** | A*0201, A*0202, A*0203 | [5] | Positive |
| **VLASTTAKA** | A*0201, A*0202, A*0203 | [4] | Negative |
| **LQSSDDFAL** | A*0201, A*0202, A*0206 | - | - |
| **TALANTIEV** | A*0201, A*0206, A*6802 | [11] | Positive |
| **ILSPLTKGI** | A*0202, A*0203, A*0206 | [4] | Positive |
| **RMVLASTTA** | A*0202, A*0203, A*0206 | [10] | Positive |
| **KTRPILSPL** | A*0202, A*0203, A*3101 | [12] | Negative |
| **QLNPIDGPL** | A*0202, A*0203, A*6802 | [13] | Positive |
| **SSFQVDCFL** | A*0202, A*0206, A*6802 | - | - |
| **LTKGILGFV** | A*0203, A*0206, A*6802 | [12] | Negative |
| **QMALQLFIK** | A*0301, A*1101, A*6801 | [5] | Positive |
| **SGRLIDFLK** | A*1101, A*3101, A*6801 | - | - |
| **RSILNTSQR** | A*1101, A*3101, A*6801 | [5] | Positive |
| **DTVNRTHQY** | A*2601, A*2902, A*6801 | [1] | Positive |
| **ITTHFQRKR** | A*3101, A*3301, A*6801 | - | - |
| **IATPGMQIR** | A*3101, A*3301, A*6801 | - | - |
| **QAGVDRFYR** | A*3101, A*3301, A*6801 | [5] | Positive |
| **HSWIPKRNR** | A*3101, A*3301, A*6801 | - | - |
| **YSHGTGTGY** | A*0101, A*2902 | [8] | Positive |
| **GILHLILWI** | A*0201, A*0206 | - | Positive |
| **MFSNKMARL** | A*0202, A*0203 | [5] | Positive |
| **NMSKKKSYI** | A*0202, A*0203 | [10] | Positive |
| **NLHIPEVCL** | A*0202, A*0203 | [13] | Positive |
| **ILGFVFTLT** | A*0202, A*0203 | [4] | Positive |
| **QMAGSSEQA** | A*0202, A*0203 | [14] | Negative |
| **QSSDDFALI** | A*0202, A*6802 | [16] | Positive |
| **SFFYRYGFV** | A*0202, A*6802 | [10] | Positive |
| **DMSIGVTVI** | A*0203, A*2902 | [10] | Positive |
| **YTMDTVNRT** | A*0203, A*6802 | [5] | Positive |
| **AVATTHSWI** | A*0203, A*6802 | [5] | Positive |
| **GTFEFTSFF** | A*0206, A*6801 | [16] | Positive |
| **TGAPQLNPI** | A*0206, A*6802 | - | - |
| **KMARLGKGY** | A*0301, A*2902 | [16] | Positive |
| **NLYNIRNLH** | A*0301, A*6801 | - | - |
| **NAISTTFPY** | A*1101, A*6801 | [16] | Negative |
| **VSILNLGQK** | A*1101, A*6801 | [5] | Positive |
| **TSFFYRYGF** | A*2902, A*6802 | [16] | Positive |
| **GYTMDTVNR** | A*3101, A*3301 | [10] | Positive |
| **HFQRKRRVR** | A*3101, A*3301 | - | - |
| **VSRARIDAR** | A*3101, A*3301 | - | - |
| **TTHFQRKRR** | A*3101, A*6801 | [10] | Positive |
| **LQLFIKDYR** | A*3101, A*6801 | [10] | Positive |
| **YRYTYRCHR** | A*3101, A*6801 | [1] | Positive |
| **FFPSSSYRR** | A*3101, A*6801 | - | - |
| **DAPFLDRLR** | A*3301, A*6801 | - | - |
| **NPLIRHENR** | A*3301, A*6801 | [4] | Negative |
| **TTAKAMEQM** | A*6801, A*6802 | [1] | Positive |

**References**

1. Harndahl M, Justesen S, Lamberth K, Roder G, Nielsen M, Buus S: **Peptide binding to HLA class I molecules: homogenous, high-throughput screening, and affinity assays.** *J Biomol Screen* 2009, **14:**173-180.
2. Assarsson E, Bui HH, Sidney J, Zhang Q, Glenn J, Oseroff C, Mbawuike IN, Alexander J, Newman MJ, Grey H, Sette A: **Immunomic analysis of the repertoire of T-cell specificities for influenza A virus in humans.** *J Virol* 2008, **82:**12241-12251.
3. Adar Y, Singer Y, Levi R, Tzehoval E, Perk S, Banet-Noach C, Nagar S, Arnon R, Ben-Yedidia T: **A universal epitope-based influenza vaccine and its efficacy against H5N1.** *Vaccine* 2009, **27:**2099-2107.
4. Hoft DF, Babusis E, Worku S, Spencer CT, Lottenbach K, Truscott SM, Abate G, Sakala IG, Edwards KM, Creech CB, et al: **Live and inactivated influenza vaccines induce similar humoral responses, but only live vaccines induce diverse T-cell responses in young children.** *J Infect Dis* 2011, **204:**845-853.
5. Squires B, Macken C, Garcia-Sastre A, Godbole S, Noronha J, Hunt V, Chang R, Larsen CN, Klem E, Biersack K, Scheuermann RH: **BioHealthBase: informatics support in the elucidation of influenza virus host pathogen interactions and virulence.** *Nucleic Acids Res* 2008, **36:**D497-503.
6. Gianfrani C, Oseroff C, Sidney J, Chesnut RW, Sette A: **Human memory CTL response specific for influenza A virus is broad and multispecific.** *Hum Immunol* 2000, **61:**438-452.
7. Matsui M, Kohyama S, Suda T, Yokoyama S, Mori M, Kobayashi A, Taneichi M, Uchida T: **A CTL-based liposomal vaccine capable of inducing protection against heterosubtypic influenza viruses in HLA-A*02:01 transgenic mice.** *Biochem Biophys Res Commun* 2010, **391:**1494-1499.
8. Alexander J, Bilsel P, del Guercio MF, Marinkovic-Petrovic A, Southwood S, Stewart S, Ishioka G, Kotturi MF, Botten J, Sidney J, et al: **Identification of broad binding class I HLA supertype epitopes to provide universal coverage of influenza A virus.** *Hum Immunol* 2010, **71:**468-474.
9. de Bree GJ, Heidema J, van Leeuwen EM, van Bleek GM, Jonkers RE, Jansen HM, van Lier RA, Out TA: **Respiratory syncytial virus-specific CD8+ memory T cell responses in elderly persons.** *J Infect Dis* 2005, **191:**1710-1718.
10. Heiny AT, Miotto O, Srinivasan KN, Khan AM, Zhang GL, Brusic V, Tan TW, August JT: **Evolutionarily conserved protein sequences of influenza a viruses, avian and human, as vaccine targets.** *PLoS One* 2007, **2:**e1190.
11. Zhong W, Reche PA, Lai CC, Reinhold B, Reinherz EL: **Genome-wide characterization of a viral cytotoxic T lymphocyte epitope repertoire.** *J Biol Chem* 2003, **278:**45135-45144.
12. Toebes M, Coccoris M, Bins A, Rodenko B, Gomez R, Nieuwkoop NJ, van de Kasteele W, Rimmelzwaan GF, Haanen JB, Ovaa H, Schumacher TN: **Design and use of conditional MHC class I ligands.** *Nat Med* 2006, **12:**246-251.
13. Ishizuka J, Grebe K, Shenderov E, Peters B, Chen Q, Peng Y, Wang L, Dong T, Pasquetto V, Oseroff C, et al: **Quantitating T cell cross-reactivity for unrelated peptide antigens.** *J Immunol* 2009, **183:**4337-4345.
14. Nijman HW, Houbiers JG, Vierboom MP, van der Burg SH, Drijfhout JW, D'Amaro J, Kenemans P, Melief CJ, Kast WM: **Identification of peptide sequences that potentially trigger HLA-A2.1-restricted cytotoxic T lymphocytes.** *Eur J Immunol* 1993, **23:**1215-1219.
15. Harndahl M, Rasmussen M, Roder G, Buus S: **Real-time, high-throughput measurements of peptide-MHC-I dissociation using a scintillation proximity assay.** *J Immunol Methods* 2010.
16. Peters B, Sidney J, Bourne P, Bui HH, Buus S, Doh G, Fleri W, Kronenberg M, Kubo R, Lund O, et al: **The immune epitope database and analysis resource: from vision to blueprint.** *PLoS Biol* 2005, **3:**e91.
