# Supplementary material for: EpicCapo: epitope prediction using combined information of amino acid pairwise contact potentials and HLA-peptide contact site information
Source: BMC Bioinformatics. 2012 Nov 24;13:313. doi: 10.1186/1471-2105-13-313 (PMC3548761; doi:10.1186/1471-2105-13-313)
Supplement: Additional file 5 — Reference and added pMHC contact sites for HLA. [file 1471-2105-13-313-S5.doc]

## Additional file 5 - Reference and added pMHC contact sites for HLA.

|  | | **Reference HLA position** | **Added HLA position** |
| --- | --- | --- | --- |
| **Nonapeptide position** | 1 | 5 59 62 63 66 163 167 171 | 7 9 45 58 67 164 |
|  | 2 | 7 9 22 24 34 45 63 66 67 70 | 99 159 |
|  | 3 | 97 99 152 155 156 159 | 9 66 67 70 160 |
|  | 4 | 65 66 155 | 62 158 |
|  | 5 | 70 73 74 97 116 155 156 | 65 69 72 114 147 150 151 152 |
|  | 6 | 66 69 70 73 74 97 114 151 155 | 65 99 147 152 156 |
|  | 7 | 97 114 147 150 152 155 | 59 63 116 133 146 |
|  | 8 | 72 73 76 80 146 | 77 147 |
|  | 9 | 77 80 81 84 95 116 123 124 143 147 | 26 33 55 58 97 142 146 |
