# Supplementary material for: EpicCapo: epitope prediction using combined information of amino acid pairwise contact potentials and HLA-peptide contact site information
Source: BMC Bioinformatics. 2012 Nov 24;13:313. doi: 10.1186/1471-2105-13-313 (PMC3548761; doi:10.1186/1471-2105-13-313)
Supplement: Additional file 6 — The scaling of positional scoring matrices. [file 1471-2105-13-313-S6.doc]

## Additional file 6 - The positional scoring matrix of EpicCapo used in the experiment that compared peptide-encoding schemes.

| **Amino acid** | **Nonapeptide position** | | | | | | | | |
| --- | --- | --- | --- | --- | --- | --- | --- | --- | --- |
|  | **1** | **2** | **3** | **4** | **5** | **6** | **7** | **8** | **9** |
| A | 6.053 | 4.651 | 5.843 | 5.812 | 5.220 | 4.497 | 5.295 | 5.243 | 7.083 |
| C | 4.474 | 1.000 | 4.359 | 4.272 | 6.514 | 6.112 | 4.928 | 5.263 | 5.066 |
| D | 2.093 | 5.074 | 5.887 | 6.432 | 5.417 | 5.575 | 4.861 | 3.889 | 5.322 |
| E | 2.030 | 5.322 | 2.958 | 6.692 | 4.146 | 4.726 | 4.936 | 5.784 | 5.322 |
| F | 8.630 | 5.220 | 6.617 | 5.082 | 6.286 | 6.680 | 7.071 | 5.993 | 3.289 |
| G | 5.468 | 3.333 | 4.632 | 5.658 | 5.579 | 3.862 | 3.349 | 6.088 | 4.841 |
| H | 4.189 | 5.646 | 4.217 | 4.636 | 6.329 | 4.900 | 4.896 | 4.411 | 4.841 |
| I | 5.650 | 7.336 | 5.883 | 5.425 | 6.021 | 7.158 | 6.550 | 4.612 | 8.295 |
| K | 6.704 | 6.676 | 2.950 | 5.437 | 4.213 | 3.388 | 2.303 | 5.263 | 5.236 |
| L | 5.705 | 9.443 | 6.664 | 4.647 | 5.457 | 6.558 | 6.416 | 6.088 | 8.157 |
| M | 6.436 | 10.000 | 7.478 | 4.861 | 5.670 | 6.242 | 6.124 | 4.008 | 5.204 |
| N | 4.604 | 1.754 | 5.086 | 5.492 | 4.458 | 5.678 | 4.892 | 5.488 | 5.322 |
| P | 2.457 | 5.982 | 4.525 | 5.247 | 3.668 | 5.034 | 6.266 | 6.116 | 4.474 |
| Q | 5.437 | 6.254 | 5.405 | 4.793 | 5.271 | 6.439 | 5.157 | 4.943 | 4.904 |
| R | 5.239 | 2.891 | 4.418 | 4.813 | 4.261 | 3.451 | 3.211 | 5.200 | 3.436 |
| S | 5.611 | 4.095 | 5.729 | 5.863 | 4.529 | 5.149 | 5.397 | 6.428 | 5.764 |
| T | 5.425 | 5.611 | 4.486 | 5.492 | 4.193 | 6.155 | 5.101 | 4.861 | 5.512 |
| V | 6.017 | 6.345 | 5.382 | 5.622 | 5.575 | 6.218 | 5.997 | 3.858 | 9.980 |
| W | 5.871 | 4.497 | 7.020 | 5.168 | 6.621 | 3.487 | 7.351 | 6.246 | 2.271 |
| Y | 8.358 | 5.322 | 6.909 | 5.011 | 7.016 | 5.137 | 6.345 | 6.672 | 2.121 |
