# Supplementary material for: EpicCapo: epitope prediction using combined information of amino acid pairwise contact potentials and HLA-peptide contact site information
Source: BMC Bioinformatics. 2012 Nov 24;13:313. doi: 10.1186/1471-2105-13-313 (PMC3548761; doi:10.1186/1471-2105-13-313)
Supplement: Additional file 7 — The positional scoring matrix of EpicCapo used in the experiment that compared peptide-encoding schemes. [file 1471-2105-13-313-S7.pdf]

Additional file 7 - The scaling of positional scoring matrices.

In this study, the original and scaled positional scoring matrices are denoted by *T* and *T*'. The (*i*, *j*)th elements of *T* and *T'* represent preferences of amino acid *i* at position *j* in nonapeptides, and are denoted by $T_{i,j}$ and $T'_{i,j}$, respectively. We simply scale the original matrix *T* into *T*' as follows:

$$T'_{i,j} = 9 \times \frac{(T_{i,j} - MIN)}{(MAX - MIN)} + 1,$$

where MAX and MIN represent the maximum and minimum values in the matrix, respectively. The example of matrix scaling is shown below.

$T_{i,j}$

MAX = 1.185

MIN = -1.095

Amino acid	Nonapeptide position 1	2	3	4	5	6	7	8	9
A	0.185	-0.170	0.132	0.124	-0.026	-0.209	-0.007	-0.020	0.446
C	-0.215	**-1.095**	-0.244	-0.266	0.302	0.200	-0.100	-0.015	-0.065
D	-0.818	-0.063	0.143	0.281	0.024	0.064	-0.117	-0.363	0.000
E	-0.834	0.000	-0.599	0.347	-0.298	-0.151	-0.098	0.117	0.000
F	0.838	-0.026	0.328	-0.061	0.244	0.344	0.443	0.170	-0.515
G	0.037	-0.504	-0.175	0.085	0.065	-0.370	-0.500	0.194	-0.122
H	-0.287	0.082	-0.280	-0.174	0.255	-0.107	-0.108	-0.231	-0.122
I	0.083	0.510	0.142	0.026	0.177	0.465	0.311	-0.180	0.753
K	0.350	0.343	-0.601	0.029	-0.281	-0.490	-0.765	-0.015	-0.022
L	0.097	1.044	0.340	-0.171	0.034	0.313	0.277	0.194	0.718
M	0.282	**1.185**	0.546	-0.117	0.088	0.233	0.203	-0.333	-0.030
N	-0.182	-0.904	-0.060	0.043	-0.219	0.090	-0.109	0.042	0.000
P	-0.726	0.167	-0.202	-0.019	-0.419	-0.073	0.239	0.201	-0.215
Q	0.029	0.236	0.021	-0.134	-0.013	0.283	-0.042	-0.096	-0.106
R	-0.021	-0.616	-0.229	-0.129	-0.269	-0.474	-0.535	-0.031	-0.478
S	0.073	-0.311	0.103	0.137	-0.201	-0.044	0.019	0.280	0.112
T	0.026	0.073	-0.212	0.043	-0.286	0.211	-0.056	-0.117	0.048
V	0.176	0.259	0.015	0.076	0.064	0.227	0.171	-0.371	1.180
W	0.139	-0.209	0.430	-0.039	0.329	-0.465	0.514	0.234	-0.773
Y	0.769	0.000	0.402	-0.079	0.429	-0.047	0.259	0.342	-0.811

$T'_{i,j}$

$$T'_{i,j} = 9 \times \frac{(T_{i,j} - MIN)}{(MAX - MIN)} + 1$$

$$T'_{1,1} = 9 \times \frac{(0.185 - (-1.095))}{(1.185 - (-1.095))} + 1$$

$= 6.053$

$$T'_{2,1} = 9 \times \frac{((-0.215) - (-1.095))}{(1.185 - (-1.095))} + 1$$

$= 4.474$

⋮

Amino acid	Nonapeptide position 1	2	3	4	5	6	7	8	9
A	6.053	4.651	5.843	5.812	5.220	4.497	5.295	5.243	7.083
C	4.474	1.000	4.359	4.272	6.514	6.112	4.928	5.263	5.066
D	2.093	5.074	5.887	6.432	5.417	5.575	4.861	3.889	5.322
E	2.030	5.322	2.958	6.692	4.146	4.726	4.936	5.784	5.322
F	8.630	5.220	6.617	5.082	6.286	6.680	7.071	5.993	3.289
G	5.468	3.333	4.632	5.658	5.579	3.862	3.349	6.088	4.841
H	4.189	5.646	4.217	4.636	6.329	4.900	4.896	4.411	4.841
I	5.650	7.336	5.883	5.425	6.021	7.158	6.550	4.612	8.295
K	6.704	6.676	2.950	5.437	4.213	3.388	2.303	5.263	5.236
L	5.705	9.443	6.664	4.647	5.457	6.558	6.416	6.088	8.157
M	6.436	10.000	7.478	4.861	5.670	6.242	6.124	4.008	5.204
N	4.604	1.754	5.086	5.492	4.458	5.678	4.892	5.488	5.322
P	2.457	5.982	4.525	5.247	3.668	5.034	6.266	6.116	4.474
Q	5.437	6.254	5.405	4.793	5.271	6.439	5.157	4.943	4.904
R	5.239	2.891	4.418	4.813	4.261	3.451	3.211	5.200	3.436
S	5.611	4.095	5.729	5.863	4.529	5.149	5.397	6.428	5.764
T	5.425	5.611	4.486	5.492	4.193	6.155	5.101	4.861	5.512
V	6.017	6.345	5.382	5.622	5.575	6.218	5.997	3.858	9.980
W	5.871	4.497	7.020	5.168	6.621	3.487	7.351	6.246	2.271
Y	8.358	5.322	6.909	5.011	7.016	5.137	6.345	6.672	2.121
